# Pyrazinamide induced thrombocytopenia

**DOI:** 10.4103/0253-7613.64495

**Published:** 2010-04

**Authors:** Surya Kant, Sanjay Kumar Verma, Vaibhav Gupta, Sunish C. Anand, Rajendra Prasad

**Affiliations:** Department of Pulmonary Medicine, Charapati Sahuji Maharaj Medical University, (Erstwhile King George‧s Medical University), Lucknow, Uttar Pradesh, India; 1 G.S.V.M. Medical College, Kanpur, UP, India

**Keywords:** Pyrazinamide, thrombocytopenia, adverse drug reaction

## Abstract

Thrombocytopenia is an uncommon but potentially life-threatening complication of certain antitubercular drugs and is characterized by rapid destruction of platelets whenever offending drug is taken by a susceptible person. We report a case of pyrazinamide-induced thrombocytopenia in a patient receiving anti tubercular drugs.

## Introduction

Most of the antitubercular drugs have side effects but serious reactions to these drugs are not common. Thrombocytopenia is an uncommon but potentially life-threatening complication of certain antitubercular drugs and is characterized by rapid destruction of platelets whenever the offending drug is taken by the susceptible person.[1] To the best of our knowledge, thrombocytopenia secondary to pyrazinamide is found to be an uncommon clinical entity. The occurrence of isolated thrombocytopenia in a patient taking several medications presents a challenging clinical problem. Laboratory confirmation of drug-induced thrombocytopenia at the time of initial presentation is not possible because tests for drug-dependent antiplatelet antibodies are not available in most laboratories. The diagnosis of drug-induced thrombocytopenia can be supported only by resolution of thrombocytopenia after discontinuation of therapy with the suspected drug.

## Case Report

A 20-year-old male student, non smoker, weighing 44 kg, was referred to Department of Pulmonary Medicine as a case of tubercular meningitis with complaints of fever, decreased appetite and headache for last 1 month, and purpuric rashes all over body for last 4 days. The patient had received streptomycin (0.7 g, i.m.), rifampicin (450 mg, p.o.) isoniazid (300 mg, p.o.) ethambutol (800 mg, p.o.), and pyrazinamide (1500 mg, p.o.) daily, over the previous 14 days.

There was no history of tuberculosis in the family, no history of drug reaction or allergic diathesis. A provisional diagnosis of rifampicin-induced thrombocytopenia was made. On general and physical examination, the patient was found having multiple, discrete, erythematous rashes all over the body. There was no peripheral lymphadenopathy or clubbing. Vital signs were normal. Routine investigations showed hemoglobin 8 g%, total leukocyte count 8,800/mm^3^ and normal differential count, renal and liver function tests. The bleeding time was 7 min and clotting time was 13 min. The platelet count was 40,000/mm^3^. The patient was seronegative for HIV, HBsAg, and the Widal test. The test for antinuclear antibodies was negative. Platelet-associated antigen tests were recommended, but the patient refused to take them due to the expenses involved. The Mantoux test was strongly positive with 22 mm induration. The patient was managed conservatively. Purpuric rashes subsided and platelet count reached to 4.5 lac/mm^3^, after 12 days. On 13th day, after informed consent, antituberculosis therapy was restarted with inj. streptomycin and tab isoniazid. We avoided rifampicin initially since we were fairly certain that it was the causal agent in the present case for thrombocytopenia. The monitoring of platelet count for next 2 days revealed no abnormality. On third day, tab ethambutol was started at a low test dose along with other drugs. The platelet counts were normal.

On 17^th^ day, a trial of pyrazinamide was given with challenging doses of 500 mg, once in a day and the patient deteriorated again with a platelet count of 80,000/mm^3^. Pyrazinamide was stopped, while the other drugs (streptomycin, isoniazid and ethambutol) were continued. On 19^th^ day, rifampicin was introduced along with streptomycin, isoniazid, and ethambutol. The monitoring of platelet count revealed no abnormality. Thus, pyrazinamide was probably the casual drug for thrombocytopenia (Naranjo scale: 6). It was not administered, thereafter. The patient was on antitubercular treatment (streptomycin, isoniazid, ethambutol, and rifampicin) when discharged and after 2 months of treatment the patient was asymptomatic and had a normal bleeding profile.

## Discussion

The identification of isolated thrombocytopenia in a patient taking several medications presents a challenging clinical problem. The causes of thrombocytopenia include viral infections, immune disorders, collagen vascular diseases, lymphoproliferative disorders, and drugs.[2] Drug-induced thrombocytopenia can be caused by quinidine, sulfonamides, chemotherapeutic agents, penicillin, barbiturates, heparin, digoxin, and estrogen. No antituberculous drug is without side effects but adverse reactions are rarely life threatening.[3] Thrombocytopenia is a well-known complication following use of these and is characterized by a rapid lowering of the platelet count in sensitive individuals. Generally, the most common offending agent for the causation of thrombocytopenia secondary to antituberculous drugs is rifampicin. A literature search revealed a total of about 35 cases of rifampicin-induced thrombocytopenia.

A review of 247 cases of drug-induced thrombocytopenia revealed that among these 9% had major bleeding (0.8% were fatal), 28% had minor bleeding, 39% had trivial bleeding, and 24% had no bleeding symptoms despite a low platelet count.[4] The 9% rate of major bleeding associated with drug-induced thrombocytopenia suggests that the problem is clinically important. However, in practice the rate may be lower. Rifampicin can occasionally cause thrombocytopenia and purpura with or without other symptoms of abnormal bleeding when used intermittently.[5,6]

Thrombocytopenia can also occur as hematological reactions following administration of pyrazinamide and isoniazid.[7] There are also case reports of thrombocytopenia due to ethambutol possibly involving an immunological mechanism.[8] Streptomycin is very rarely implicated as a cause of thrombocytopenia. Kant *et al*, reported thrombocytopenia secondary to rifampicin, ethambutol, and pyrazinamide in a single individual.[9] Drug-induced thrombocytopenia is assessed by using standard criteria to define three levels of severity of bleeding.[4]

The diagnosis of drug-induced thrombocytopenia can be supported only by the resolution of thrombocytopenia after discontinuation of therapy with the suspected drug.[10] Laboratory confirmation of drug-induced thrombocytopenia at the time of initial presentation is not possible because tests for drug-dependent antiplatelet antibodies are not available in most clinical laboratories. Furthermore, the safety of continuing treatment with a suspected drug when results of drug-dependent antibody tests are negative has not been established. While restarting antitubercular therapy in a patient suspected of having drug-induced thrombocytopenia, it is prudent to start three drugs not given previously including one parenteral agent (aminoglycoside or polypeptide) and two oral agents that are safe especially when a life-threatening adverse drug reaction has occurred. Rechallenge with the offending drug even in small doses is contraindicated if purpura occurs.[11] Platelet count should be monitored regularly and platelets should be transfused to maintain levels above 20000/mm_3_. Corticosteroids are of no benefit in drug-induced thrombocytopenia. Plasmapheresis can be helpful in some cases. Offending drug should not be reused as only a minute quantity of drug is needed to trigger this adverse drug reaction.
